# Vegetative growth and cluster development in Shiraz grapevines subjected to partial root-zone cooling

**DOI:** 10.1093/aobpla/plt036

**Published:** 2013-08-13

**Authors:** Suzy Y. Rogiers, Simon J. Clarke

**Affiliations:** 1 NSW Department of Primary Industries, Locked Bag 588, Wagga Wagga, NSW 2678, Australia; 2 National Wine and Grape Industry Centre, Wagga Wagga, NSW, Australia; 3 Faculty of Science, Charles Sturt University, Wagga Wagga, NSW, Australia

**Keywords:** Berry composition, grapevine, Shiraz, shoot growth, soil temperature, split pot, *Vitis vinifera*.

## Abstract

Heterogeneity in root-zone temperature both vertically and horizontally may contribute to the uneven vegetative and reproductive growth often observed across vineyards. An experiment was designed to assess whether the warmed half of a grapevine root zone could compensate for the cooled half in terms of vegetative growth and reproductive development. We divided the root system of potted Shiraz grapevines bilaterally and applied either a cool or a warm treatment to each half from budburst to fruit set. Shoot growth and inflorescence development were monitored over the season. Simultaneous cooling and warming of parts of the root system decreased shoot elongation, leaf emergence and leaf expansion below that of plants with a fully warmed root zone, but not to the same extent as those with a fully cooled root zone. Inflorescence rachis length, flower number and berry number after fertilization were smaller only in those vines exposed to fully cooled root zones. After terminating the treatments, berry enlargement and the onset of veraison were slowed in those vines that had been exposed to complete or partial root-zone cooling. Grapevines exposed to partial root-zone cooling were thus delayed in vegetative and reproductive development, but the inhibition was greater in those plants whose entire root system had been cooled.

## Abstract (precis)

Grapevine roots can be exposed to a range of temperatures at any particular moment because the root system can explore large volumes of soil over great depths and distances. A split-pot experiment was designed to assess how vegetative and reproductive development respond to partial and whole root-zone warming following winter dormancy. Simultaneous cooling and warming of parts of the root system slowed shoot elongation, leaf expansion and berry development compared to plants with a fully warmed root-zone, but not to the same extent as those with a fully cooled root-zone.

## Introduction

Plant roots can be exposed to a range of temperatures because root systems can explore large volumes of soil over great depths and distances. The temperature of this soil is dependent on its depth below the surface, composition and moisture, as well as the degree of exposure to sun and wind in relation to aspect, slope and barriers (Kerridge *et al*. 2013). In grapevines, non-uniform irrigation and heterogeneous soil type across a vineyard lead to inconsistent vegetative growth, yield and berry composition (Trought 1997; Bramley *et al*. 2011). Simultaneous cool and warm root-zone microclimates may be an additional source of this variability.

Springtime soil temperatures are important to grapevine canopy development following dormancy because the preliminary stages of growth are dependent on carbohydrate mobilization from the roots (Yang and Hori 1979; Yang *et al*. 1980). Cool root zones following winter dormancy in perennial crops can delay carbohydrate reserve mobilization, effectively slowing shoot growth and inflorescence development (Greer *et al*. 2006; Field *et al*. 2009; Rogiers *et al*. 2011). Once leaves become net exporters of carbon, cool root zones can also have a direct effect on vegetative and reproductive growth by lowering net assimilation rates. This is a consequence of either alterations in photosynthetic reactions (Cai and Dang 2002; Erice *et al*. 2006) or decreased stomatal conductance (Wan *et al*. 1999, 2004; Rogiers and Clarke 2013). A reduction in fruit set (the proportion of flowers that become berries) is a common result of this lowered net assimilation (Candolfi-Vasconcelos *et al*. 1994; Caspari *et al*. 1998; Lebon *et al*. 2008; Rogiers *et al*. 2011).

Split-root studies have been used in grapevines to examine the effects of partial root-zone drying (PRD) on grapevine physiology and fruit composition (Dry *et al*. 2000; Santos *et al*. 2003; Souza *et al*. 2003; Antolín *et al*. 2006). In PRD, the opposing halves of the root zone are alternately exposed to wet and dry cycles by applying or withholding irrigation. This irrigation system decreases vegetative vigour while maintaining plant water status and improves leaf and whole-plant water-use efficiency (Dry *et al*. 1996; Stoll *et al*. 2000). Partial root-zone drying can advance berry ripening through decreased competition with vegetative growth, increased light penetration into the fruiting zone and modified vine signalling (Santos *et al*. 2003; Antolín *et al*. 2006; Bindon *et al*. 2007, 2008). Because whole root-zone cooling can limit leaf expansion (Rogiers and Clarke 2013), partial root-zone cooling, comparable with PRD, may result in a similar inhibition of vegetative growth with repercussions on reproductive development.

The relative proportions of plant signals or growth regulators such as abscisic acid, cytokinins or gibberellins released by cooled and warmed roots (Atkin *et al*. 1973; Zhang *et al*. 2008; Field *et al*. 2009) and perceived by expanding buds probably affect shoot and leaf development. However, the delayed vegetative growth that results from roots exposed to cool soil may be overcome by those parts of the root system growing in a warmer segment of the soil. The warmed portion of the root system could, for instance, produce enough signal to drive cell division and expansion so that vegetative growth is equivalent to that of fully warmed roots. Alternatively, growth may be directly proportional to the volume of roots that is exposed to the warm soil. Nutrient and water uptake by plants as well as root carbohydrate mobilization might be reduced when half of the root system is exposed to cool soil and this could result in an intermediate amount of shoot growth. Using a split-pot method, we examined grapevine response to root zones 5 °C above and below the average vineyard soil temperature found at a depth of 15 cm in a warm grape-growing region of Australia. The objective of this work was to clarify how vegetative growth and reproductive development respond to partial root-zone cooling.

## Methods

### Root-zone temperature treatments

**Figure 1.**
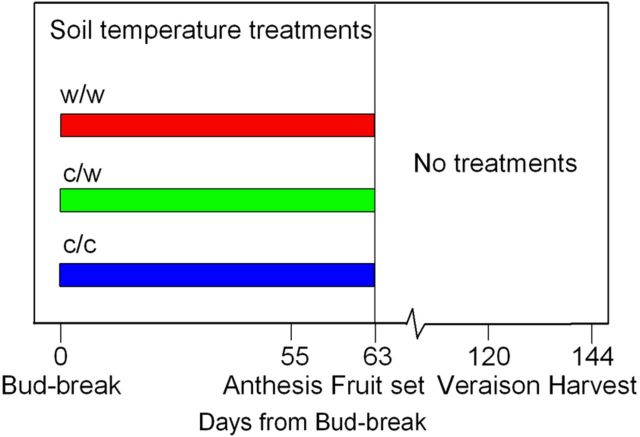
Timeline of treatment applications and major phenological events.

Twenty dormant Shiraz grapevines (*Vitis vinifera*, clone PT23), 4 years of age, growing in 15-L containers were pruned to two spurs, each carrying two buds. Immediately thereafter they were replanted using a premium commercial potting mix into 15-L (25 cm in diameter, 30 cm in height) split pots that had been halved bilaterally using a heavy plastic partition insulated on both sides with 10-mm dense foam. A heat-exchange system consisting of 13-mm polyethylene tubing was inserted into each half of the pot and coiled within the root mass of the plant. Details of the heat-exchange system are given elsewhere (Rogiers and Clarke 2013). The pots were placed outdoors in a bird-proof enclosure in a randomized complete block design in one row oriented in the north to south direction. Two months later, three root-zone temperature treatments (*n* = 5) consisting of cool/cool (c/c), cool/warm (c/w) and warm/warm (w/w) were applied from the onset of budburst (E-L stage 5, Eichhorn and Lorenz 1977) to the termination of fruit set (stages 27–29) for a total of 63 days (see Fig. 1 for timeline of treatments relative to phenological stages). For the remainder of the season, the root-zone temperature was allowed to fluctuate diurnally according to ambient conditions. The plants were irrigated twice daily using an automated irrigation system with drippers inserted into both halves of the pot. Excess water was allowed to drain from the pot.

Thermocouples (TT-T-36, Omega Engineering, Northmead, NSW, Australia) were inserted into the centre of the root mass in both halves of one pot and logged at 30-min intervals (CR1000, Campbell Scientific, Townsville, Australia). In addition, manual measurements were carried out to a 15-cm depth twice weekly with a hand-held thermometer. Soil temperature averaged 17.7/17.8 °C for the c/c, 18.0/28.0 °C for the c/w and 28.4/29.6 °C for the w/w treatment. Soil moisture was measured twice weekly on both halves with an ML2x Theta Probe (Delta-T Devices, Cambridge, UK) inserted to a depth of 15 cm. Soil moisture averaged 21/19 % for the c/c, 20/17 % for the c/w and 18/19 % for the w/w treatment. A weather station was placed at canopy height within the trial. It was equipped with an air temperature probe (Intercap HMP50, Vaisala, Hawthorn, VIC, Australia) logging at half-hourly intervals and recorded an average minimum of 10.3 °C and a maximum of 28.5 °C over the duration of the experiment. A quantum sensor (Apogee, SQ-110, Logan, UT, USA), also logging at half hourly intervals, recorded an average daily maximum photosynthetically active radiation of 1200 µmol m^−2^ s^−1^. Thermocouples were placed at cluster height, 30–40 cm above the soil surface, and air temperature during the treatment period averaged 20.6 ± 0.2 °C and 20.7 ± 0.3 °C above the cool and warm soil, respectively.

### Canopy and inflorescence characteristics

Leaf length was measured weekly on each leaf opposite the basal tendril in the three-node repeating leaf/tendril arrangement. Root-zone temperature did not affect the relationship between leaf length and area, and therefore, leaf area was calculated from leaf length using an allometric relationship obtained from leaves destructively harvested from adjacent vines of the same variety and grown over the same period under the same ambient conditions. Area of these leaves was measured using a leaf area meter (LI-3000; Li-Cor, Lincoln, NE, USA) and the following calculation was applied: area (in cm^2^) = 0.00748 × length^2^ (in mm) + 0.294 × length^2^ (in mm) − 5.86. A leaf was registered as emerged on the day it reached a length of 30 mm, calculated by linear interpolation between leaf length measurements across time points. Shoot length was also measured weekly with a flexible measuring tape from the spur junction to the shoot apex. Nylon mesh bags were placed over each inflorescence just prior to anthesis and percentage fruit set was calculated by counting flower caps and aborted flowers. Berry diameter was measured with digital callipers on eight berries per cluster for all clusters. Veraison was assessed visually as first signs of colour change.

### Data analysis

An analysis of variance (GenStat release 15.0, VSN, Hertfordshire, UK) was used for comparison of treatment effects during the root-zone temperature treatments and least significant differences (LSDs) were calculated at 5 % significance. Results of the statistical analyses are presented in the tables and figures. Values presented in the text are means ± s.e.

## Results

### Root-zone temperatures

**Figure 2.**
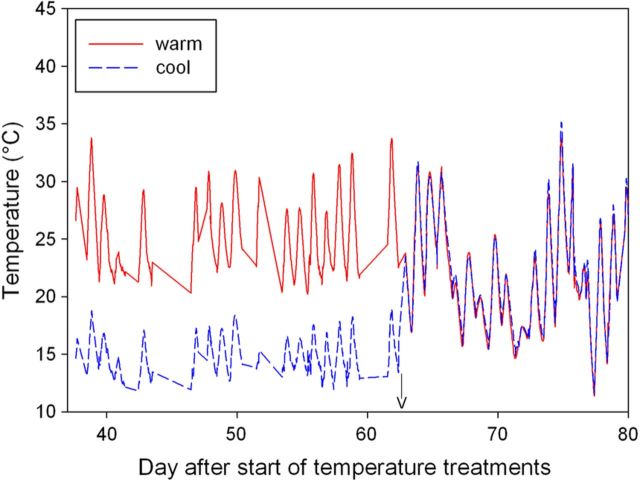
Root-zone temperature profiles of a Shiraz grapevine grown in a split pot. Half of the roots were exposed to a warm temperature while the other half were exposed to a cool temperature treatment for 63 days. Beyond this day, the root-zone temperature was not controlled.

Root-zone temperature averaged at 25 ± 0.1 °C in the warm half and 14.6 ± 0.07 °C in the cool half of the split pot over the 63-day treatment period (Fig. 2). The daily maximum temperature averaged at 28.1 ± 0.8 °C, while the daily minimum was 21.2 ± 0.3 °C in the warm half of the pot. On the cool-treated side, the daily maximum averaged at 16.2 ± 0.3 °C, while the daily minimum was 12.5 ± 0.2 °C. Therefore, the treatments produced a warm–cool contrast of about 10 °C with diurnal fluctuations between daily maximum and minimum temperatures of 7 °C in the warm treatment and 3.5 °C in the cool treatment. After the cessation of the treatments, the daily maximum averaged at 25.2 ± 0.9 °C and the daily minimum at 17.7 ± 0.6 °C with diurnal fluctuations of 7.5 °C.

### Vegetative growth

**Figure 3.**
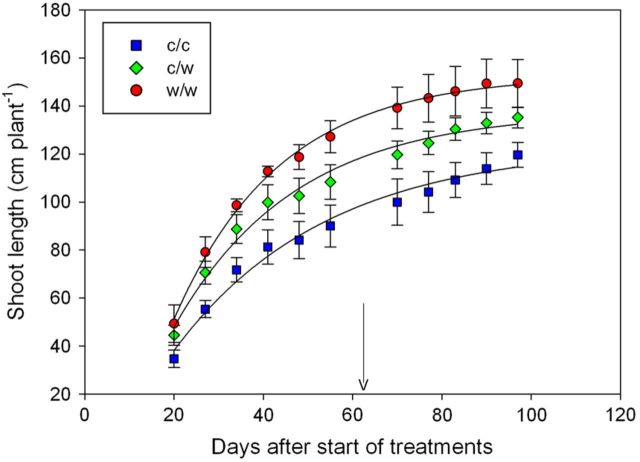
Shoot growth of Shiraz vines grown in split pots and exposed to cool/cool, cool/warm or warm/warm root-zone temperatures (mean ± s.e.). The treatments were applied from budburst to fruit set. Arrow indicates when treatments were terminated. The main factors of treatment (*P* < 0.001) and day (*P* < 0.001) were significant. Exponential curves were fitted to the data.

Budburst (E-L stage 5) varied by 4 days between plants, but was not affected by root-zone temperature (*P* = 0.86). The shoot number per vine averaged at 3.9 ± 0.08 and also did not differ between treatments (*P* = 0.26). The shoot growth was slowest in the c/c treatment and after 8 weeks of treatment (Day 55), shoot length was greater by 41 and 21 % in the w/w and c/w chambers when compared with the c/c treatments (*P* < 0.001, Fig. 3). Over the subsequent 5 weeks of uniform temperature exposure across the entire root system, shoot growth continued in all the treatments. However, despite more rapid shoot growth in the c/c vines during this latter part of the season (19.6, 15.5 and 10.2 cm over 27 days for c/c, c/w and w/w, respectively), significant differences in total plant shoot length persisted across the treatments.

**Figure 4.**
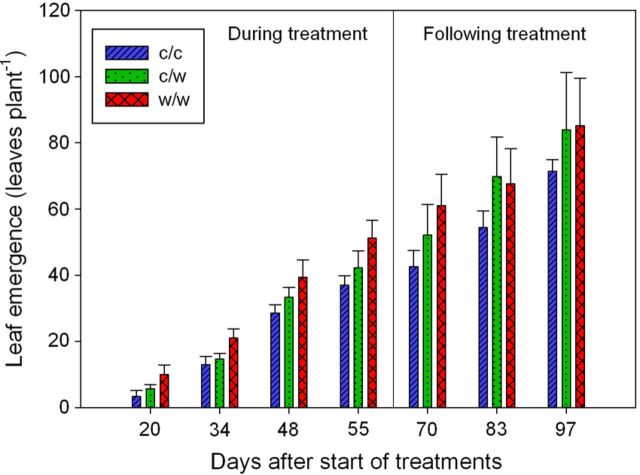
Leaf emergence of Shiraz vines grown in split pots and exposed to cool/cool, cool/warm or warm/warm root-zone temperatures (mean ± s.e.). The treatments were applied from budburst to fruit set. The main factors of treatment (*P* < 0.001) and day (*P* < 0.001) were significant during the treatment period. After the termination of the treatments only day (*P* < 0.001) was statistically significant.

Leaf appearance (equivalent to node number) was also more rapid during the w/w treatment when compared with the c/c treatment (*P* < 0.001, Fig. 4). After 20 days of treatment, plants exposed to fully cooled root zones had 63 % fewer leaves when compared with plants exposed to fully warmed root zones. Plants exposed to partial root zone warming exhibited intermediate levels of leaf emergence at budburst. Beyond Day 20, the rate of leaf emergence of the c/c treatment was not different from that of the c/w treatment (*P* = 0.51) or the w/w treatment (*P* = 0.14) and averaged at 1.0 leaves per day (*r*^2^ = 0.70). On Day 55, plant leaf number was greater by 45 and 26 % in the w/w and c/w pots when compared with the c/c treatments. After the termination of the treatments, leaf number continued to increase but was no longer statistically different between treatments (*P* > 0.05).

**Figure 5.**
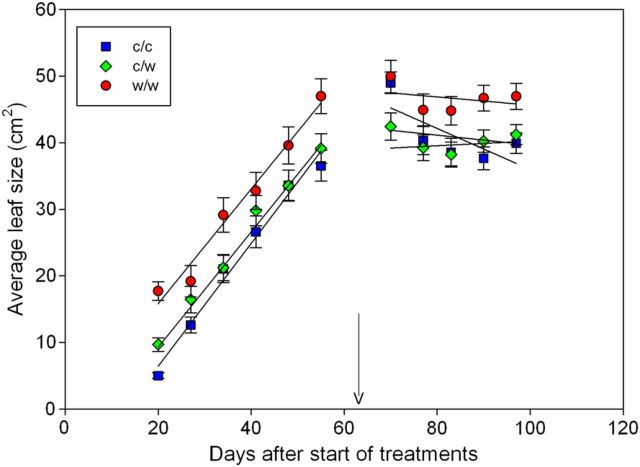
Average leaf size of Shiraz grapevines grown in split pots and exposed to cool/cool, cool/warm or warm/warm root-zone temperatures (mean ± s.e.). Included in the averages are immature and fully expanded leaves. The treatments were applied from budburst to fruit set. Arrow indicates when treatments were terminated. The main factors of treatment (*P* < 0.001) and day (*P* < 0.001) were significant. Linear curves were fitted to the data.

Like shoot growth and leaf emergence, leaf size was also affected by the root-zone treatments (*P* < 0.001, Fig. 5). Within 20 days of initiating the treatments, the leaf size (averaged across immature and fully expanded leaves) of those vines receiving the w/w treatment was 3.5-fold greater than that of the c/c treatment and 1.9-fold greater than that of the c/w treatments. Over the subsequent 5 weeks of treatment, differences between the c/c and c/w treatments were no longer apparent; however, the w/w leaves remained on average up to 1.2-fold larger than leaves of the other treatments. Following the termination of the treatments, the average leaf size remained approximately stable with the exception of the c/c treatment where it declined by 0.3 cm^2^ day^−1^ (*P* < 0.01) due to the production of smaller leaves.

**Figure 6.**
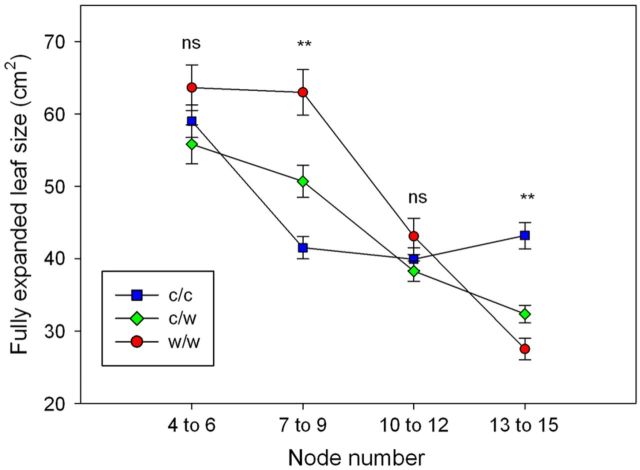
Fully expanded leaf size at nodes 4–15 for shoots of Shiraz grapevines grown in split pots and exposed to cool/cool, cool/warm or warm/warm root-zone temperatures (mean ± s.e.). The treatments were applied from budburst to fruit set. Treatment was significant at nodes 7–9 and nodes 13–15 (*P* < 0.001).

The size of fully expanded leaves located on nodes 4–6 and 10–12 was not affected by the root-zone treatments (*P* > 0.05, Fig. 6). However, at nodes 7–9 maximum leaf size was greatest for the w/w, intermediate for c/w and lowest for the c/c treatment (*P* < 0.001, 63.0, 50.7 and 41.5 cm^2^, respectively). Nodes 13–15 emerged after the treatments were terminated and the largest leaves at these positions were those from the c/c treatment (43.2 cm^2^) followed by the c/w (32.3 cm^2^) and the w/w treatment (27 cm^2^).

The total vine leaf area at the termination of the root-zone temperature treatments was 21 % greater in the c/w treatment and 51 % greater in the w/w treatment compared with the c/c treatment (Table 1). Six weeks later, these differences in total vine leaf area were still apparent but to a lesser extent (11 % greater in the c/w treatment and 29 % greater in the w/w treatment compared with the c/c treatment).

**Table 1.** Plant leaf area as a function of root-zone temperature. The treatments were applied over a 63-day period from budburst to fruit set. Different letters indicate significant differences (*P* < 0.05).

Root-zone temperature	Leaf area (cm^2^ plant^−1^)	
	Day 60	Day 120
Cool/cool	840^a^	1381^a^
Cool/warm	1014^ab^	1532^ab^
Warm/warm	1269^b^	1782^b^
LSD	281	254
*F*-test, *P*	0.04	0.03

### Anthesis, fruit set and veraison

**Figure 7.**
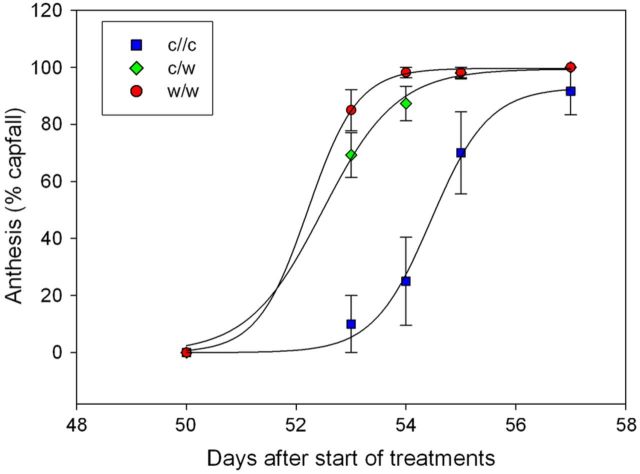
Onset of flowering expressed as percentage capfall from Shiraz inflorescences grown in split pots and exposed to cool/cool, cool/warm or warm/warm root-zone temperatures (mean ± s.e.). The treatments were applied from budburst to fruit set. The main factors of treatment (*P* < 0.001) and day (*P* < 0.001) were significant. Sigmoidal curves were fitted to the data.

Flowering proceeded over a 4- to 8-day period and was dependent on root-zone temperature (*P* < 0.001, Fig. 7). On Day 53 of the treatment period, capfall was only 10 % in the c/c treatment and 69 % in the c/w treatment, while the w/w treatment averaged at 98 %. Two days later, capfall had reached 98–100 % with the exception of the c/c treatment, which lagged behind at 70 %.

Root-zone temperature did not affect the number of inflorescences that emerged on each plant (1.5 ± 0.3, F pr = 0.91); however, flower number per inflorescence was lowest in the c/c treatment (*P* < 0.01, Table 2). Inflorescences carried on average only 38 flowers in the c/c treatment but 95–119 flowers in the other treatments with no effect of partial root-zone cooling. Average percentage fruit set was not different between the treatments and ranged from 84 to 93 % (Table 2). Approximately 2 months later, the berries entered the ripening phase of development. The onset of veraison proceeded most rapidly for the w/w treatment and on Day 3 was 14.3 % compared with 5.8 and 6.9 % for the c/c and c/w treatments, respectively (Table 3). Veraison was complete within 10 days but beyond the fifth day, the extent of berry colour change was not affected by the root-zone treatments (Table 3).

**Table 2.** Effect of root-zone temperature on mean inflorescence flower number and percentage fruit set. Shiraz vines were exposed to cool/cool, cool/warm or warm/warm root-zone temperatures from budburst to fruit set in the concurrent season. Different letters indicate significant differences (*P* < 0.05).

Root-zone temperature	Inflorescence flower number	Fruit set (%)
Cool/cool	38^a^	93^a^
Cool/warm	119^b^	84^a^
Warm/warm	95^b^	85^a^
LSD	41	13
*F*-test, *P*	0.003	0.426

**Table 3.** Percentage of berries per cluster that had reached veraison 118 and 123 days after the initiation of treatments. Shiraz vines were grown in split pots and exposed to cool/cool, cool/warm or warm/warm root-zone temperatures from budburst to fruit set. Different letters indicate significant differences (*P* < 0.05).

Root-zone temperature	% Veraison	
	Day 118	Day 123
Cool/cool	5.8^a^	84.1^a^
Cool/warm	6.9^b^	92.7^a^
Warm/warm	14.3^c^	88.6^a^
LSD	0.08	15
*F*-test, *P*	0.03	0.99

### Berry size

Berry enlargement was responsive to the root-zone temperatures that were applied until fruit set (Table 4). Prior to veraison, berry diameter was only slightly greater in the c/w pots compared with the c/c treatment; however, berries of the w/w treatment were 23 % wider than those of the c/c treatment (*P* < 0.001). At veraison, the berry diameters of the w/w treatment were still 8 % larger than those of the other treatments (*P* < 0.001).

**Table 4.** Berry diameter as a function of root-zone temperature. The treatments were applied over a 63-day period from budburst to fruit set and the berry diameter assessments were carried out 15 and 60 days after the treatments were terminated. Different letters indicate significant differences (*P* < 0.05).

Root-zone temperature	Berry diameter (mm)	
	Pre-veraison	Veraison
Cool/cool	5.35^a^	10.11^a^
Cool/warm	5.75^a^	10.08^a^
Warm/warm	6.58^b^	10.94^b^
LSD	0.38	0.30
*F*-test, *P*	0.001	0.001

The mean cluster rachis fresh weight was at least double for those vines grown under partial and complete root-zone warming compared with the c/c treatment (*P* < 0.05, Table 5). Clusters also held more than double the number of fully developed berries when root zones were exposed to c/w and w/w treatments compared with the c/c treatments (*P* < 0.05, Table 5). Despite this, the number of undeveloped berries (berries that remained small, hard and green, including live-green ovaries) was also 3- to 4-fold higher for these treatments (*P* < 0.01, Table 5).

**Table 5.** Effect of root-zone temperature on mean rachis fresh weight, developed and undeveloped berry number. Shiraz vines were exposed to cool/cool, cool/warm or warm/warm root-zone temperatures from budburst to fruit set in the concurrent season. Different letters indicate significant differences (*P* < 0.05).

Root-zone temperature	Rachis fresh weight (g)	Developed berry number/cluster	Undeveloped berry number/cluster
Cool/cool	1.33^a^	22.0^a^	6.7^a^
Cool/warm	2.71^b^	59.5^b^	26.4^b^
Warm/warm	2.71^b^	46.1^b^	17.9^b^
LSD	0.88	17.6	10.3
*F*-test, *P*	0.012	0.02	0.005

## Discussion

### Canopy development

Root-zone temperature has a key role in grapevine vegetative growth (Woodham and Alexander 1966; Skene and Kerridge 1967; Zelleke and Kliewer 1979, 1980). In our study, partially cooled root zones inhibited shoot growth but not to the same extent as fully cooled root zones. When grapevines are released from winter dormancy, the xylem delivers water, carbohydrates, nutrients, hormones and other plant signals to the buds to drive shoot elongation and leaf expansion (Andersen and Brodbeck 1988, 1989*a*; Campbell and Strother 1996; Field *et al*. 2009). Initially xylem sap is transported by the pressure created through the establishment of an osmotic gradient (Scholander *et al*. 1955; Sperry *et al*. 1987). Root pressure is temperature dependent (Andersen and Brodbeck 1989*b*) and the delivery rate of growth regulators and other metabolites to the shoots can thus be slowed if half of the root zone is cooled, consequently decreasing rates of shoot growth and development. Similarly, as leaves mature and stomata become functional, shoots of plants in a partially warmed root zone would be exposed to less root-derived signal than a fully warmed root zone due to lower leaf transpiration (Wan *et al*. 2004; Rogiers and Clarke 2013). Furthermore, the synthesis of signals in the cooled root zone may be slowed due to lower rates of temperature-dependent reactions (Cox *et al*. 2000) so that the concentration that the shoot receives is less than in a fully warmed root zone. Shoot elongation is stimulated by cytokinins (Werner *et al*. 2003) and it has been shown that warm soil can increase nucleotide cytokinin concentration in bleeding sap (Field *et al*. 2009). Similar to grapevines exposed to PRD (Dry *et al*. 2000), a partial inhibition of shoot elongation under partial root-zone cooling may be a direct effect of decreased cytokinin levels. Gibberellins are also important for shoot elongation and appear to decline in xylem exudate as root temperature declines (Atkin *et al*. 1973).

Along with shoot elongation, the rate of leaf emergence and average leaf size were reduced when vines were subjected to partially or fully cooled root zones. While the size of fully expanded leaves located on nodes 4–6 was not altered by the treatments, leaves from nodes 7–9 were largest in vines that had received the fully warmed treatment, with intermediate sizes in the partially warmed root-zone treatments. Even though the first 12 or so grapevine leaves are preformed in the bud and leaf size is ontogenetically predetermined to some extent (Parkhurst and Loucks 1972; Greer and Weston 2010), final leaf size is particularly sensitive to stresses imposed early in leaf development, during the cell proliferation and expansion phases. These stresses reduce final leaf size by reducing the rate of leaf expansion and/or the duration over which expansion occurs (Granier and Tardieu 2009). Because full root-zone cooling inhibited the expansion of leaves at nodes 7–9 more so than partial root-zone cooling, it is possible that plant signals such as abscisic acid, an inhibitor of leaf expansion (Bacon *et al*. 1998), occurred at higher concentrations in leaves when roots were fully cooled. Conversely, promoters of leaf expansion such as gibberellins and cytokinins (Engelke *et al*. 1973; Ross *et al*. 1993) may have been synthesized in the roots of the warmed half of the c/w treatment but not in the roots of the fully cooled treatment.

Carbon stress can be induced by exposing roots to cool temperatures. This is because inhibited root carbohydrate mobilization can result in low energy and structural carbon availability for expanding leaves. The roots are the major storage site of starch in grapevines and mobilization is highly dependent on the temperature of the soil (Field *et al*. 2009; Rogiers *et al*. 2011). Semillon grapevines rely on carbohydrate reserves for canopy development over the first 6 weeks after budburst (Greer *et al*. 2012). Because leaf expansion was lower in fully cooled root zones than in those that were partially cooled, perhaps minimal root carbohydrate degradation had occurred in this treatment. It is also probable that root-zone cooling decreased water uptake (BassiriRad *et al*. 1991) by decreasing hydraulic conductance (Lopez and Nobel 1991; Cochard *et al*. 2000; Wan *et al*. 2001) or inhibited root uptake of ions such as K^+^ and their accumulation within leaf cells (Luo *et al*. 2012), thereby decreasing the osmotic gradient that drives water into the cell.

Unlike those leaves from nodes 7–9, leaves located on nodes 10–12 were not different in size. Across the experiment, nodes 10–12 represent a mixed cohort, consisting of leaves formed before and after the treatment period. The relatively large size of the leaves located at nodes 13–15 of the pre-cooled plants indicates that the stress that constrained leaf size was eliminated with the termination of the treatments and some mechanism has promoted the formation of relatively large leaves. This observation also indicates that typical ontogenetic trends in leaf size on grapevine shoots (e.g. Schultz 1992) may be perturbed when the spring soil temperatures are depressed. The unusually large leaves at nodes 13–15 in the fully cooled root-zone treatment may be due to the under-utilization of the root carbohydrate pool in the period up to fruit set and the subsequent mobilization of these reserves later in the season when photoassimilates from fully functioning leaves are also a source of energy and structural carbon.

### Flower formation

Full root-zone cooling during inflorescence development decreased inflorescence flower number and delayed the onset of anthesis. Similar observations were made in Chardonnay (Rogiers *et al*. 2011) and Muscat of Alexandria grapes (Shimamura and Kubota 1978). Inflorescence initiation begins in the previous season (May 1965) and this is why inflorescence number was not affected by the root-zone treatments from budburst onwards. Furthermore, there was no treatment effect on cluster branching and this is likely because the inflorescence branch primordia were established in the previous season prior to dormancy (Srinivasan and Mullins 1981). Conversely, flower primordia are not initiated until the following spring, after the break of bud dormancy (Snyder 1933; Winkler and Shemsettin 1937; Agaoglu 1971; Srinivasan and Mullins 1981). Each branch primordium of the inflorescence primordium divides many times and, ultimately, it produces the flower initials. The treatments were applied during the development of the flower initials and this temporal overlap made it possible for the root-zone treatments to have an effect on the flower number. This mode of action may be via decreased carbohydrate supply to the developing organ through cool temperature inhibition of root carbohydrate reserve mobilization or through a reduction of recent photoassimilate supply resulting from impaired vegetative growth. Both of these sources supply carbon to early flower development (Kliewer 1975; Goffinet 2004; Lebon *et al*. 2008; Smith and Holzapfel 2009).

Root-zone exposure to cool temperatures triggers stomatal closure in grapevines (Rogiers and Clarke 2013) as well as in other species (Wan *et al*. 2004) and is hypothesized to be the result of the accumulation of abscisic acid and other root-to-shoot signals transported through the xylem sap (Tachibana 1988; Lee *et al*. 1993; Wan *et al*. 2004; Zhou *et al*. 2007). This decline in stomatal conductance is accompanied by a decrease in net photoassimilation (Wan *et al*. 1999, 2004; Rogiers and Clarke 2013). Therefore, fewer flowers in cool root zones may initially be the result of lower availability of mobilized carbohydrate reserves but subsequently due to reduced photoassimilates as a result of reduced stomatal conductance, smaller functional leaf area and altered partitioning to favour carbohydrate reserve replenishment. A close relationship between carbohydrate status and flower development exists in a number of woody plants (Rodrigo *et al*. 2000).

### Anthesis and berry development

Aside from decreased flower number, the progression of flower opening was markedly slowed in vines with fully cooled root zones compared with fully warmed root zones. The dynamic analysis of anthesis revealed a 5-day delay in the ability of flowers grown on fully cooled vines to reach 90 % capfall, but inflorescences of partially cooled root zones had only a 1-day delay in flower opening. Compared with partial root-zone cooling, full root-zone cooling may result in a more severe delay of hormone production that favours flower development. Alternatively, complete root-zone cooling may decrease to a greater extent both assimilate and water transport to the inflorescence so that flower development is slowed. Flowering, like many other developmental processes, is dependent on cardinal air temperature and thermal sum but soil temperature may be an additional driver for these processes (Craufurd and Wheeler 2009).

Whole and partial root-zone cooling until fruit set delayed berry expansion over the following weeks as well as the onset of veraison. This may have been simply a carry-on effect from the delayed flowering period. Additionally, the smaller leaf size of plants grown in partial or whole root-zone cooling likely decreased whole vine carbon assimilation, further delaying the development of the reproductive organs. It is also possible that these vines allocated energy towards root growth after the release from the cool temperature stress (Pregitzer *et al*. 2000; Wan *et al*. 2001), and thus, the availability of photoassimilates for berry development likely declined since roots and reproductive growth often compete for carbohydrates (Hansen 1977; Eissenstat and Duncan 1992). The ability of root-zone temperature to delay or accelerate the onset of ripening may have important implications for the planning of vineyard activities such as pesticide spray regimes and scheduling of harvest dates.

## Conclusions

Heterogeneity in soil temperature from budburst to fruit set at the plant scale is strongly expressed in the vegetative and reproductive development that takes place in this period. Partial root-zone cooling elicited intermediate responses in terms of leaf emergence and shoot growth. The effects of soil temperature up to fruit set continued to be evident over the remainder of the season. Depression of vegetative growth prior to fruit set was followed by increases in the rate of shoot elongation and the size of newly emerged leaves. The onset of flowering and veraison was delayed by several days in vines exposed to full root-zone cooling, but this was less severe in those vines exposed to partial root-zone cooling. These observations suggest that warming of one half of the root system did not fully compensate for cooling in the other half. As a result, spatial variations in soil temperature from budburst to fruit set have the potential to produce heterogeneity in cluster phenology, an attribute used to schedule many vineyard activities.

## Sources of Funding

This work was supported by the grape growers and winemakers of Australia through their investment body, the Grape and Wine Research Development Corporation, with matching funds from the federal government.

## Contributions by the Authors

S.Y.R. and S.J.C. designed and conducted the experiment and analysed the data. S.Y.R. wrote the manuscript and S.J.C. provided significant editorial comments.

## Conflict of Interest Statement

None declared.
